# Supplementary figures and images for: Enhancing reproducibility in stable isotope analysis (SIA) of fish eye lenses: A comparison between lamina number and diameter
Source: PLoS One. 2025 Jun 26;20(6):e0326345. doi: 10.1371/journal.pone.0326345 (PMC12200824; doi:10.1371/journal.pone.0326345)

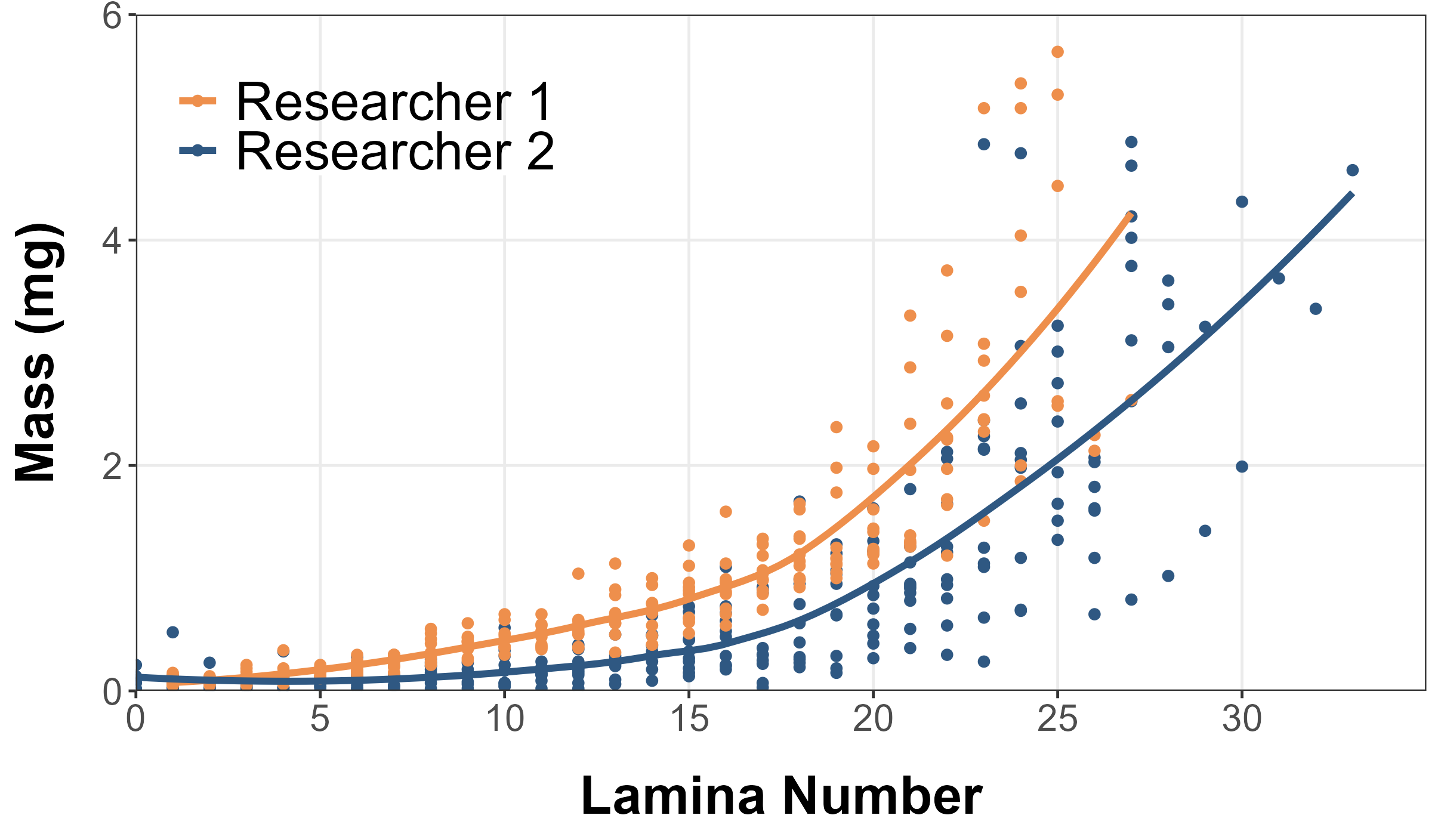

Supplement: S1 Fig — Laminae were weighed using a microbalance. Researcher 1 (orange) peeled fewer and heavier lamina on average. Researcher 2, however, tended to peel lighter lamina, resulting in more lamina. (TIFF) [file pone.0326345.s001.tiff]

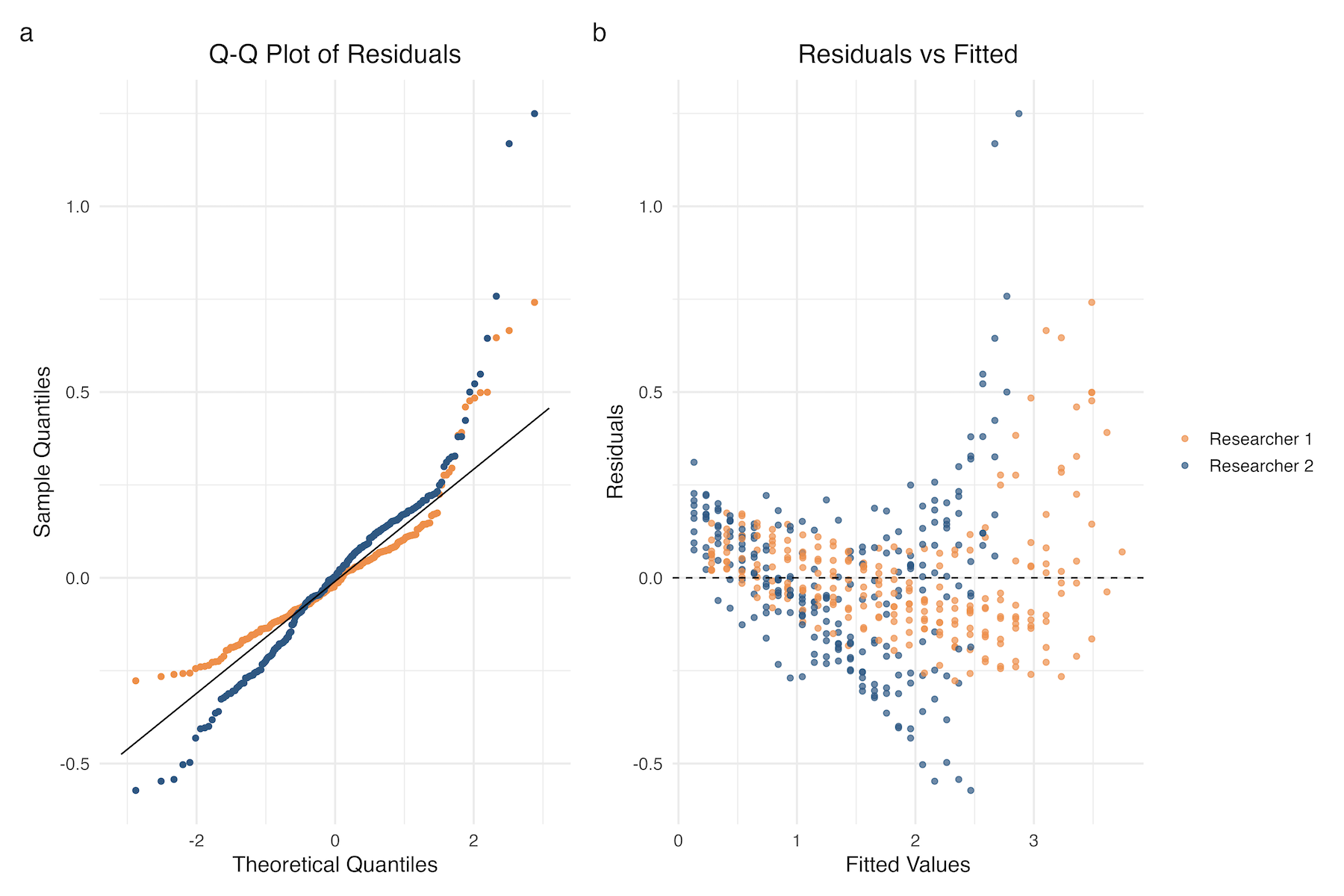

Supplement: S2 Fig — (a) Q-Q plot of residuals colored by researcher. Mild deviations from normality were observed at the tails. (b) Residuals versus fitted values colored by researcher. An increase in variance was observed across fitted values, indicative of heteroscedasticity and confirmed by the Breusch-Pagan test (BP = 71.931, p < 0.001). (TIFF) [file pone.0326345.s003.tiff]
