# Supplementary material for: Enhancing reproducibility in stable isotope analysis (SIA) of fish eye lenses: A comparison between lamina number and diameter
Source: PLoS One. 2025 Jun 26;20(6):e0326345. doi: 10.1371/journal.pone.0326345 (PMC12200824; doi:10.1371/journal.pone.0326345)
Supplement: S1 Table — Summary of the assumption tests for the two-sample t-test comparing lamina count between researchers. Non-significant p values (p > 0.05) suggest normal distribution within each group and equality of variance between groups, satisfying the assumptions for a standard t-test. (PDF) [file pone.0326345.s002.pdf]

S1 Table: Assumption testing for the two-sample t-test comparing lamina counts between researchers.

Test	Group(s)	Statistics	p-value	Interpretation
Shapiro –Wilk Normality	Researcher 1	W = 0.927	0.807	Normal Distribution
Shapiro –Wilk Normality	Researcher 2	W = 0.962	0.422	Normal Distribution
Levene’s Test Equal Variance	Researcher 1 vs 2	F = 0.262	0.615	Equal Variance
