# Supplementary material for: Enhancing reproducibility in stable isotope analysis (SIA) of fish eye lenses: A comparison between lamina number and diameter
Source: PLoS One. 2025 Jun 26;20(6):e0326345. doi: 10.1371/journal.pone.0326345 (PMC12200824; doi:10.1371/journal.pone.0326345)
Supplement: S2 Table — Researcher 2 was the reference group. Robust standard errors (HC3) were applied to correct for heteroscedasticity. Significant predictors are indicated by p-values, with *** denoting p < 0.001. (PDF) [file pone.0326345.s004.pdf]

S2 Table: Linear regression model results for lens diameter as a function of lamina number and researcher.

Term	***Estimate***	***Robust SE***	***t-value***	***p-value***
(Intercept)	0.1310	0.0261	5.02	7.11e-07 ***
Lamina number	0.1016	0.0028	36.23	<2.2e-16 ***
Researcher 1 vs 2	0.1482	0.0305	4.85	1.64e-06 ***
Lamina number x Researcher 1	0.0268	0.0033	8.03	7.15e-15 ***
