# Supplementary material for: Enhancing reproducibility in stable isotope analysis (SIA) of fish eye lenses: A comparison between lamina number and diameter
Source: PLoS One. 2025 Jun 26;20(6):e0326345. doi: 10.1371/journal.pone.0326345 (PMC12200824; doi:10.1371/journal.pone.0326345)
Supplement: S1 File — Supplemental information on methods. (PDF) [file pone.0326345.s005.pdf]

Supplemental Information

Eye lens peeling notes

S1 Text: Supplemental information on methods

1. In some adult lenses used in this study, the outermost area occasionally includes an outer cortex or the partially formed laminae, which contains living cellular organelles [17]. The inconsistency in observing this lamina may be attributed to the freezing and thawing storage process of the whole eye, as well as the subsequent lens extraction procedure.

2. In our study, we found that the lens has four main quadrants that can be peeled off given adequate moisture. Excessive moisture can cause too much of the lamina to be peeled off at once, while insufficient moisture can make the lens peeling difficult, causing the four main quadrants to split into multiple pieces. With the right balance of moisture, multiple discrete laminae can be removed from the lens, allowing for higher-resolution isotope data to be interpreted from each individual fish.
