# Supplementary material for: Enhancing reproducibility in stable isotope analysis (SIA) of fish eye lenses: A comparison between lamina number and diameter
Source: PLoS One. 2025 Jun 26;20(6):e0326345. doi: 10.1371/journal.pone.0326345 (PMC12200824; doi:10.1371/journal.pone.0326345)
Supplement: Attachment — Submitted filename: PONE-D-24-57172_review_final.pdf [file pone.0326345.s006.pdf]

Review of PONE-D-24-57172: “Enhancing reproducibility in stable isotope analysis (SIA) of fish eye lenses: A comparison between lamina number and diameter”.

General comment:

The manuscript presents a well-structured study comparing two methods for analysing fish eye lens using stable isotope analysis (SIA). The study is relevant and timely, given the increasing use of eye lenses for dietary and life-history reconstructions. The methodology is clear, and the results convincingly demonstrate that using lens diameter instead of lamina number improves reproducibility. Its findings have practical implications for standardising ecological research. However, some areas require some clarification, additional justification, or minor revisions.

Minor comments:

Line 20: The sentence "Fish eye lenses grow sequentially throughout their ontogeny, resulting in a structure of multiple layers, or laminae" is accurate but slightly awkward. Suggestion: Rephrase to "Fish eye lenses grow throughout their ontogeny, forming multiple sequential layers, or laminae," to improve readability.

Line 22: The analogy "much like tree rings" is useful but lacks context for readers unfamiliar with SIA. Suggestion: Add a brief clarification, e.g., "much like tree rings, which record environmental conditions over time."

Line 26: The phrasing "peeled lenses from each eye of the same adult Chinook Salmon" is unclear about the experimental design. Suggestion: Clarify as "Each researcher independently delaminated one lens from each of 10 adult Chinook Salmon."

Line 33: "Analyzing the laminae based on lamina number resulted in significant variability between researchers" lacks specificity. Suggestion: Specify the nature of variability, e.g., "Analysis based on lamina number showed significant variability in isotope values and lamina counts between researchers."

Line 40: The opening sentence is maybe a bit abrupt. Suggestion: Start with a broader context, e.g., "Understanding animal diets is fundamental to ecology, informing conservation and management strategies, yet capturing lifetime dietary shifts remains challenging."

Lines 40-47: This first paragraph could use some references.

Line 48: The claim about eye lenses as an "emerging archival tissue" is true and could be elaborated. Suggestion: Add "due to their metabolically inert laminae, which preserve stable isotope values reflective of diet over time."

Line 65: “or isotope maps” instead of “or geographic map”.

Line 68: The example of the Atlantic goliath grouper is relevant but long/wordy. Suggestion: "For example, SIA of eye lenses revealed habitat shifts in Atlantic goliath grouper (*Epinephelus itajara*), emphasizing the role of mangrove connectivity in conservation [11]."

Line 81: Microscopes’ cameras do provide good resolution to measure eye lens diameters. However, are they always needed if there is no access to costly microscope cameras? The use

of a digital calliper (that is not as expensive as a microscope's camera) is also a valuable tool to measure eye lens diameters.

Line 102: Fish origin is not mentioned. Add context if available.

Line 105: What temperature were the Eppendorf tubes kept at?

Line 106: The delamination process is a bit unclear regarding lens assignment. Suggestion: Revise to "Both researcher independently delaminated one lens (left or right) from each of the 10 fish, ensuring paired lenses from the same fish were analyzed separately."

Line 107: Citation accuracy should be verified. Suggestion: Ensure [6] directly supports the delamination protocol or if another reference related to delamination could be added. Additional, reverse citing order i.e., [5], then [6].

Lines 107-108 and Lines 238-239: "A membrane of gelatinous material, often referred to as the outer cortex" has already been defined in the introduction. You can start this sentence by: "The outer cortex..."

Line 112: how were the laminae dried? What temperature, for how long?

Line 116: "meet the 0.6mg-6 mg" 6 mg seems a lot for stable isotope measurements. Is this correct?

Line 121: Figure 3 and its description are well presented. This is a very valuable section. However, most of the caption description could be incorporated into the main text.Line 129: T-tests are suitable for comparing the means of two independent groups (the researchers). However, there is no statement about the fact that the data are normally distributed, and variances are equal, which is a prerequisite to use these tests.

Line 174: "Figures 7a, 7b, 7C, and 7d" could be changed to (Figs 7a–d) for clarity.

Line 186: no need to redefine SIA.

Line 193: "and are used in combination with other endogenous records like otoliths to provide a comprehensive understanding of the species being studied" – any references here?

Line 195: From there, the whole discussion section could use some references. For instance, citing the studies who are already using eye lens diameter.

Line 210: As comment above, lens diameter is presented as a novel proposal, though prior studies used it. Suggestion: Adjust to "Lens diameter, already adopted in some studies [references], proved more reliable than lamina number."

Line 237: Future Works - This section is brief and relevant. Any thoughts on developing a standardised method to measure the outer cortex?

Line 252: in the methods, it reads that the stable isotope lab used was: "All samples were then submitted to the CAMAS Stable Isotope Laboratory at Idaho State University for $\delta^{13}C$ and $\delta^{15}N$ analysis" – which is it?
