# Supplementary material for: Enhancing reproducibility in stable isotope analysis (SIA) of fish eye lenses: A comparison between lamina number and diameter
Source: PLoS One. 2025 Jun 26;20(6):e0326345. doi: 10.1371/journal.pone.0326345 (PMC12200824; doi:10.1371/journal.pone.0326345)
Supplement: Attachment — Submitted filename: PONE-D-24-57172.pdf [file pone.0326345.s007.pdf]

PLOS ONE

Enhancing reproducibility in stable isotope analysis (SIA) of fish eye lenses: A comparison between lamina number and diameter

--Manuscript Draft--

**Manuscript Number:**	PONE-D-24-57172
**Article Type:**	Research Article
**Full Title:**	Enhancing reproducibility in stable isotope analysis (SIA) of fish eye lenses: A comparison between lamina number and diameter
**Short Title:**	Methods to maximize reproducibility of stable isotope diet chronologies using fish eye lenses
**Corresponding Author:**	Danhong Ally Li<br>University of California Davis<br>Davis, CA UNITED STATES OF AMERICA
**Keywords:**	Fish eye lens; stable isotope; chinook salmon; diet reconstruction; food web; ecology; fisheries science; oncorhynchus tshawytscha; salmon
**Abstract:**	Analyzing stable isotopes in archival tissues, such as fish eye lenses, is used to document shifts in feeding ecology, diet, habitat use, and to reconstruct life history. Fish eye lenses grow sequentially throughout their ontogeny, resulting in a structure of multiple layers, or laminae. These laminae represent the chronology of the fish's life, much like tree rings. Lenses are protein-rich, which makes them an ideal structure for analyzing light isotopes such as $\delta^{13}C$, $\delta^{15}N$, and δ34S. These light isotopes are primarily integrated into the lens tissue through the fish's diet. As research begins to emerge using eye lenses to reconstruct the life histories of fishes, the need for a reproducible method of delamination grows. For this study, two different researchers peeled lenses from each eye of the same adult Chinook Salmon (Oncorhynchus tshawytscha) (n=10 fish). Lens lamina number, diameter (mm), and mass (mg) of each lamina were recorded. Laminae were then submitted for isotopic analysis of both $\delta^{13}C$ and $\delta^{15}N$. Isotope values were used as a validation to compare delamination patterns between researchers. $\delta^{13}C$ and $\delta^{15}N$ values from the lenses were then plotted using both the assigned lamina number and lens diameter to compare the difference between researchers. Analyzing the laminae based on lamina number resulted in significant variability between researchers. However, when lens diameter was used instead of lamina number, isotope patterns throughout the lenses of the same fish were nearly identical. Using lens diameter removes subjectivity between researchers, thereby increasing the reproducibility of the technique and providing a more robust interpretation of the data.
**Order of Authors:**	Alexandra Chu
	Danhong Ally Li
	Miranda Bell-Tilcock
	Miranda Lowe-Webb
	Carson Jeffres
	Rachel C. Johnson
**Additional Information:**	
**Question**	**Response**
**Financial Disclosure**<br><br>Enter a financial disclosure statement that describes the sources of funding for the work included in this submission. Review the submission guidelines for detailed requirements. View published research	Yes

Powered by Editorial Manager® and ProduXion Manager® from Aries Systems Corporation

articles from *PLOS ONE* for specific examples.<br><br>This statement is required for submission and **will appear in the published article** if the submission is accepted. Please make sure it is accurate.<br><br>**Funded studies**<br>Enter a statement with the following details:<br>• Initials of the authors who received each award<br>• Grant numbers awarded to each author<br>• The full name of each funder<br>• URL of each funder website<br>• Did the sponsors or funders play any role in the study design, data collection and analysis, decision to publish, or preparation of the manuscript?<br><br>Did you receive funding for this work?	
Please add funding details.<br>as follow-up to "**Financial Disclosure**<br><br>Enter a financial disclosure statement that describes the sources of funding for the work included in this submission. Review the submission guidelines for detailed requirements. View published research articles from *PLOS ONE* for specific examples.<br><br>This statement is required for submission and **will appear in the published article** if the submission is accepted. Please make sure it is accurate.	Funding for this project was provided by California Department of Fish and Wildlife Proposition 1 agreement number P1896030.<br>PIs: Carson Jeffres and Rachel Johnson.

Powered by Editorial Manager® and ProduXion Manager® from Aries Systems Corporation

**Funded studies**<br>Enter a statement with the following details:<br>• Initials of the authors who received each award<br>• Grant numbers awarded to each author<br>• The full name of each funder<br>• URL of each funder website<br>• Did the sponsors or funders play any role in the study design, data collection and analysis, decision to publish, or preparation of the manuscript?<br><br>Did you receive funding for this work?"	
Please select the country of your main research funder (please select carefully as in some cases this is used in fee calculation).<br>as follow-up to "**Financial Disclosure**<br><br>Enter a financial disclosure statement that describes the sources of funding for the work included in this submission. Review the submission guidelines for detailed requirements. View published research articles from *PLOS ONE* for specific examples.<br><br>This statement is required for submission and **will appear in the published article** if the submission is accepted. Please make sure it is accurate.<br><br>**Funded studies**<br>Enter a statement with the following details:<br>• Initials of the authors who received each award<br>• Grant numbers awarded to each author<br>• The full name of each funder<br>• URL of each funder website<br>• Did the sponsors or funders play any role in the study design, data collection and analysis, decision to publish, or preparation of the manuscript?<br><br>Did you receive funding for this work?"	UNITED STATES - US
**Competing Interests**	NO authors have competing interests

Use the instructions below to enter a competing interest statement for this submission. On behalf of all authors, disclose any competing interests that could be perceived to bias this work—acknowledging all financial support and any other relevant financial or non-financial competing interests.<br><br>This statement is required for submission and **will appear in the published article** if the submission is accepted. Please make sure it is accurate and that any funding sources listed in your Funding Information later in the submission form are also declared in your Financial Disclosure statement.<br><br>View published research articles from *PLOS ONE* for specific examples.<br><br>**NO authors have competing interests**<br><br>Enter: *The authors have declared that no competing interests exist.*<br><br>**Authors with competing interests**<br><br>Enter competing interest details beginning with this statement:<br><br>*I have read the journal's policy and the authors of this manuscript have the following competing interests: [insert competing interests here]*<br><br>* typeset	
**Ethics Statement**<br><br>Enter an ethics statement for this submission. This statement is required if the study involved:<br><br>• Human participants<br>• Human specimens or tissue<br>• Vertebrate animals or cephalopods<br>• Vertebrate embryos or tissues	Samples used in this paper were remnant tissues collected as a part of the USFWS Adult Chinook Salmon Sampling Plan.

Powered by Editorial Manager® and ProduXion Manager® from Aries Systems Corporation

• Field research<br><br>Write "N/A" if the submission does not require an ethics statement.<br><br>General guidance is provided below. Consult the submission guidelines for detailed instructions. **Make sure that all information entered here is included in the Methods section of the manuscript.**	

Powered by Editorial Manager® and ProduXion Manager® from Aries Systems Corporation

**Format for specific study types**<br><br>**Human Subject Research (involving human participants and/or tissue)**<br>• Give the name of the institutional review board or ethics committee that approved the study<br>• Include the approval number and/or a statement indicating approval of this research<br>• Indicate the form of consent obtained (written/oral) or the reason that consent was not obtained (e.g. the data were analyzed anonymously)<br><br>**Animal Research (involving vertebrate animals, embryos or tissues)**<br>• Provide the name of the Institutional Animal Care and Use Committee (IACUC) or other relevant ethics board that reviewed the study protocol, and indicate whether they approved this research or granted a formal waiver of ethical approval<br>• Include an approval number if one was obtained<br>• If the study involved *non-human primates*, add *additional details* about animal welfare and steps taken to ameliorate suffering<br>• If anesthesia, euthanasia, or any kind of animal sacrifice is part of the study, include briefly which substances and/or methods were applied<br><br>**Field Research**<br><br>Include the following details if this study involves the collection of plant, animal, or other materials from a natural setting:<br>• Field permit number<br>• Name of the institution or relevant body that granted permission	
**Data Availability**<br><br>Authors are required to make all data underlying the findings described fully available, without restriction, and from the time of publication. PLOS allows rare exceptions to address legal and ethical concerns. See the PLOS Data Policy and FAQ for detailed information.	Yes - all data are fully available without restriction

Powered by Editorial Manager® and ProduXion Manager® from Aries Systems Corporation

<table>
<tr>
<td>A Data Availability Statement describing where the data can be found is required at submission. Your answers to this question constitute the Data Availability Statement and will be published in the article, if accepted.

Important: Stating 'data available on request from the author' is not sufficient. If your data are only available upon request, select 'No' for the first question and explain your exceptional situation in the text box.

Do the authors confirm that all data underlying the findings described in their manuscript are fully available without restriction?</td>
<td></td>
</tr>
<tr>
<td>Describe where the data may be found in full sentences. If you are copying our sample text, replace any instances of XXX with the appropriate details.

• If the data are held or will be held in a public repository, include URLs, accession numbers or DOIs. If this information will only be available after acceptance, indicate this by ticking the box below. For example: All XXX files are available from the XXX database (accession number(s) XXX, XXX.).
• If the data are all contained within the manuscript and/or Supporting Information files, enter the following: All relevant data are within the manuscript and its Supporting Information files.
• If neither of these applies but you are able to provide details of access elsewhere, with or without limitations, please do so. For example:

Data cannot be shared publicly because of [XXX]. Data are available from the XXX Institutional Data Access / Ethics Committee (contact via XXX) for researchers who meet the criteria for access to confidential data.

The data underlying the results presented in the study are available from (include the name of the third party</td>
<td>The data will be held in DRYAD, a public data repository. The data submission is currently in draft with DOI: 10.5061/dryad.8cz8w9h1s; It can only be submitted once a manuscript number is received. Thus, file containing metadata of all samples used in this study will be available only after acceptance.</td>
</tr>
</table>

and contact information or URL).<br>• This text is appropriate if the data are owned by a third party and authors do not have permission to share the data.<br><br>* typeset	
Additional data availability information:	Tick here if the URLs/accession numbers/DOIs will be available only after acceptance of the manuscript for publication so that we can ensure their inclusion before publication.

Powered by Editorial Manager® and ProduXion Manager® from Aries Systems Corporation

Manuscript

Click here to access/download;Manuscript;Lens Methods Finalized Script.docx

Long Title: Enhancing reproducibility in stable isotope analysis (SIA) of fish eye lenses: A comparison between lamina number and diameter

Short Title: Methods to maximize reproducibility of stable isotope diet chronologies using fish eye lenses

Alexandra Chu[1⁋], Danhong Ally Li[1⁋]*, Miranda Bell-Tilcock[2], Miranda Lowe-Webb[1], Carson Jeffres[1], Rachel C. Johnson[1,3]

[1] Center for Watershed Sciences, University of California, Davis, California, United States of America

[2] Delta Stewardship Council, Sacramento, California, United States of America

[3] National Marine Fisheries Service, Southwest Fisheries Science Center, Santa Cruz, California, United States of America

*Corresponding authors

Email: dhli@ucdavis.edu (Danhong Ally Li)

⁋These authors contributed equally to this work.

Abstract

Analyzing stable isotopes in archival tissues, such as fish eye lenses, is used to document shifts in feeding ecology, diet, habitat use, and to reconstruct life history. Fish eye lenses grow sequentially throughout their ontogeny, resulting in a structure of multiple layers, or laminae. These laminae represent the chronology of the fish's life, much like tree rings. Lenses are protein-rich, which makes them an ideal structure for analyzing light isotopes such as $\delta^{13}C$, $\delta^{15}N$, and $\delta^{34}S$. These light isotopes are primarily integrated into the lens tissue through the fish's diet. As research begins to emerge using eye lenses to reconstruct the life histories of fishes, the need for a reproducible method of delamination grows. For this study, two different researchers peeled lenses from each eye of the same adult Chinook Salmon (*Oncorhynchus tshawytscha*) (n=10 fish). Lens lamina number, diameter (mm), and mass (mg) of each lamina were recorded. Laminae were then submitted for isotopic analysis of both $\delta^{13}C$ and $\delta^{15}N$. Isotope values were used as a validation to compare delamination patterns between researchers. $\delta^{13}C$ and $\delta^{15}N$ values from the lenses were then plotted using both the assigned lamina number and lens diameter to compare the difference between researchers. Analyzing the laminae based on lamina number resulted in significant variability between researchers. However, when lens diameter was used instead of lamina number, isotope patterns throughout the lenses of the same fish were nearly identical. Using lens diameter removes subjectivity between researchers, thereby increasing the reproducibility of the technique and providing a more robust interpretation of the data.

Introduction

Understanding animal diets is crucial not only for unraveling the ecological roles of species but also for identifying key regions and habitats that support biodiversity. Dietary analysis provides insights into food web dynamics, predator-prey relationships, and ecosystem health. Investigating dietary shifts is particularly important for species with complex life cycles that utilize different food sources throughout their development. Capturing a complete dietary history is challenging, especially for migratory species, as traditional methods like prey sampling and gut content analysis offer only a snapshot of an individual's diet at a specific time.

Fish eye lenses are an emerging archival tissue that allows us to trace diet history throughout an individual’s life [1]. Eye lenses consist of three distinct zones: the gelatinous living outer cortex, the hardened intermediate zone and the dense inner core [2] (Fig 1). As the fish matures, each lamina becomes metabolically inert after its formation, preserving the isotopic composition of the fish’s diet during that time (Fig 2A). This layered structure makes it the ideal tool for tracing dietary information throughout an individual’s life [3] (Fig 2B).

Fig 1. Illustrations of Chinook Salmon (*Oncorhynchus tshawytscha*) eye lenses.

Fig 2. Pictures of Chinook Salmon (*Oncorhynchus tshawytscha*) eye lenses. (A) Image of the Chinook Salmon eye lens without the outer cortex taken on a Leica S9i

with the accompanying software, LAS X. (B) Cross section image of a Chinook Salmon lens mounted in resin and taken on ImagePro scientific image analysis software.

Stable isotope analysis (SIA) of fish eye lenses has been conducted across different species, with studies investigating ontogenetic shifts, resource partitioning, and trophic histories [4–6]. SIA provides a powerful tool for studying animal diet histories, movement patterns, and natal origins without requiring the subsequent recapture of the same individual [7,8]. This method has been successfully used to create isoscapes, or geographic maps that depict unique isotopic values or food web patterns across the landscape [9,10]. Stable Isotope Analysis of recovered tissues is especially useful when studying elusive species that are difficult to tag. For instance, SIA of eye lenses has been utilized to trace habitat changes through dietary changes in species like the Atlantic goliath grouper (*Epinephelus itajara*), highlighting their use of the mangroves while also emphasizing the importance of conservation efforts that focus on seascape connectivity, since protecting only one habitat might not be sufficient to protect this species [11]. Similarly, by combining SIA of eye lenses with a mixing model, researchers have discovered that Clear Lake Hitch (*Lavinia exilicauda chi*) transition from benthic to pelagic food sources, pinpointing the specific size at which this transition occurs [1]. In addition to fishes, $\delta^{13}C$ and $\delta^{15}N$ laminae values of neon flying squid (*Ommastrephes bartramii*) were used to compare dietary shifts across the life stages in both eastern and western stocks [12].

In previous studies, lenses have been represented using both lamina number as well as diameter for SIA analysis [1,3,10]. When studying eye lenses, lamina number is

a more accessible metric to use compared to lens diameter. Measuring the lens necessitates specialized and often costly equipment like microscope cameras. However, the lamina number is assigned by the researcher and is therefore heavily influenced by each researcher's tendencies. Thus, interpreting the data using lamina number can lead to errors, especially if there are multiple researchers processing samples. While most studies have shifted to using lens diameter over lamina number, presumably for reasons of consistency and accuracy, none have explicitly evaluated the need for this shift. This study addresses this existing gap in the literature by examining and quantifying inconsistencies in assigning lamina number among researchers, providing a clearer understanding of the reliability of lens diameter as a metric, and offering recommendations for improving the methodology in this field.

In order to compare the reproducibility between using lamina number and using diameter method for delamination, two researchers worked on one of two lenses from the same fish without communication. A total of 10 fish were analyzed for this study. Lamina number, diameter (mm) and mass (mg) were assigned, measured, and recorded as metrics to test the consistency and reproducibility between researchers. Lastly, laminae were then submitted for isotopic analysis for both $\delta^{13}C$ and $\delta^{15}N$ so the difference in trends occurred between different individuals across metrics could be compared. This study strives to offer a refined and reproducible method for lens delamination by reducing the intrinsic human differences in the delamination process.

Methods

Lens technique

Lenses were extracted from both eyes of 10 adult fall-run Chinook Salmon (*Oncorhynchus tshawytscha*). Lenses were extracted by creating an incision in the eyes and then removed with a pair of forceps. Each lens was then stored in a 1.5 mL Eppendorf tube and frozen until delamination could take place.

Both researchers delaminated one of the two lenses from the same fish, for all 10 fish used in this study, using previously established methods [6] and [5]. A membrane of gelatinous material, often referred to as the outer cortex [2,13], was observed on the surface of most lenses and carefully removed by rolling them on clean Kimwipes. Then individual laminae were peeled until the core was reached (Fig 3). After each lamina was removed, the lens was measured to the nearest thousandth of a millimeter using a Leica S9i imaging microscope with LAS X v5.0.3 imaging software. Laminae were then stored in pre-weighed 8x5mm tin capsules (Elemental Microanalysis pressed tin capsules) and once dried, were weighed to the nearest hundredth of a milligram on a Mettler Toledo Semi-Micro Balance (MS105DU). Individual lamina that exceeded the target weight (> 6.0 mg) were homogenized and subsampled to meet the 0.6mg-6 mg dried weight criteria for $\delta^{13}C$ and $\delta^{15}N$ analysis. All samples were then submitted to the CAMAS Stable Isotope Laboratory at Idaho State University for $\delta^{13}C$ and $\delta^{15}N$ analysis. Individual researchers worked independently to ensure their methods remained unbiased.

Fig 3. Pictures of lens delamination process. (A) The lens was positioned with one of the poles facing upward, where the laminae visibly converge. (B) An individual lamina was removed in a pedal-to-pedal motion peeling from the pole, with intermittent

hydration using a small amount of DI water to facilitate separation. (C) The lamina was separated into four quadrants after thoroughly peeling around the entire lens with its pole side still facing upward. (D) All materials of the lamina were separated and gathered aside for storage in a tin capsule for SIA submission.

Statistical analysis

To quantify the patterns and differences between researchers, a t-test was used to examine the difference in the average number of laminae generated per person in R [14]. The average width of each lamina was calculated by subtracting the lens diameters measured before and after the lamina was removed. The difference of lamina width measurements between the two researchers was assessed, with the aim of identifying potential patterns or trends under the assumption of even growth in the left and right eye from the same fish. To quantify the observations in varying thickness of lamina, a linear regression model was constructed using researcher and lamina width as factors in an analysis of variance (ANOVA). Significance level of $P = < 0.05$ was used to determine the significance of researchers in the differences of lamina width at the identical lamina number. Patterns in lamina counts, diameter, and isotope values were compared visually between readers using the dplyr and ggplot2 packages in R [14]. The four fish with the largest discrepancy in lamina number between researchers were used to display the variation in data.

Results

Between-researcher variation

Researchers 1 and 2 displayed distinct variations in their peeling methods. Notably, Researcher 1 exhibited a tendency to peel thicker laminae compared to Researcher 2, resulting in a mean total lamina count of 24 (+/- 2.1), while Researcher 2, who tended to peel thinner laminae, achieved a higher mean total lamina count of 27.7 (+/- 2.7) (see Figures 4 and 5). Researcher 1's total lamina count did not exceed 26, whereas Researcher 2 acquired a maximum of 33 laminae. A t-test found there to be a statistically significant difference in the average lamina number produced per eye between the two researchers ($t = 3.33$, $P < 0.05$).

Fig 4. Box plot displaying differences in total laminae between researchers. The box denotes the median and interquartile range, and the whiskers extend to 1.5 times the interquartile range.

Fig 5. Box plot showing average lamina widths for each lamina between researchers. The box denotes the median and interquartile range, and the whiskers denote 1.5 times the interquartile range.

Researcher 1 consistently recorded larger lens diameters than Researcher 2 for each of the identical laminae numbers. Similar to larger diameters, researcher 1 removed laminae with greater mass than researcher 2 for the same lamina number on average (see Fig. S1, supplemental). The ANOVA from the linear regression model showed that both researchers presented distinct trends in their peeling methods that significantly influenced the assigned lamina number for the same lens diameter ($F(1, 500) = 134.71$, $P < 2.2e-16$, Table S1 in Supplemental File). Due to between researcher

variation linked with lamina number assignment, the same lamina number might correspond to different lens diameters for fish delaminated by different researchers, thus causing a mismatch with isotope values (Fig 6).

Figure 6: Scatter plot of lamina number plotted against the lens diameter (mm) by researcher.

Using isotope values for validation

A visual comparison was then performed using $\delta^{13}C$ and $\delta^{15}N$ values from two lenses of the same fish. These plots compared the utilization of assigned lamina numbers and measured lens diameters as metrics, as shown in Figures 7a, 7b, 7c, and 7d. The $\delta^{13}C$ and $\delta^{15}N$ values served as grounding references for assessing the relative accuracy between lamina number and lens diameter. When plotting the $\delta^{13}C$ and $\delta^{15}N$ trajectories against lens diameter, a much more consistent alignment between the two researchers was evident (Figs. 7c and 7d). This suggests that the discrepancy between the isotope trajectories is due to the use of lamina number as a metric.

Figure 7. Plots of $\delta^{13}C$ and $\delta^{15}N$ values of 4 individuals against lamina number and lens diameter (mm). (A) $\delta^{13}C$ values from consecutive laminae plotted against lamina number. (B) $\delta^{15}N$ values from consecutive laminae plotted against lamina number. (C) $\delta^{15}N$ values from consecutive laminae plotted against lens diameter (mm). (D) $\delta^{15}N$ values from consecutive laminae plotted against lens diameter (mm).

Discussion

Stable isotope analysis (SIA) of fish eye lenses has become increasingly popular for interpreting a fish's life history [3], offering insights into diet [10] and habitat use [1,15]. This tool, combined with other endogenous records like otoliths, offers a comprehensive understanding of fish life histories, which is essential for addressing population-level needs. As research in this area grows, the need for standardized methods to ensure reproducibility becomes more crucial. This is particularly important since eye lens isotopes are interpreted and correlated to other metrics, such as fork length [4,12,16], and are used in combination with other endogenous records like otoliths to provide a comprehensive understanding of the species being studied.

In the study of otoliths, researchers count annuli to age fish. This method is well-established in otolith microchemistry analysis, leading ecologists to naturally gravitate towards applying similar techniques to eye lens analysis. However, unlike otolith analysis, which reveals a full profile of annuli through grinding or sectioning, eye lens analysis involves peeling laminae off the lens, which does not result in a readable profile. The count of lamina is more variable than that of annuli since, without reference to the full profile, the definition or boundary between each lamina can be ambiguous. Therefore, caution is recommended when adapting otolith methodologies to eye lens studies. Plotting lamina number against lens diameter highlights differences in peeling tendencies between researchers (see Fig 6).

This discrepancy arises because each researcher's subjective interpretation of where one lamina ends, and another begins can lead to inconsistent results. Although the isotopic trends of $\delta^{13}C$ and $\delta^{15}N$ are expected to align between eyes, when lamina

number is used as the parameter on the x-axis there is a persistent lag in isotopic trajectories between researchers (Figs 7a and 7b) [3].

Using lamina number to interpret the isotopic profile of these fish makes it challenging to extract valuable conclusions when focusing solely on analyzing a specific life stage (see Figs 7a and 7b). Given these complexities, lens diameter was proposed as an alternative metric for processing and analyzing fish eye lens isotope data. When the $\delta^{13}C$ and $\delta^{15}N$ values were plotted using lens diameter instead of lamina number, the trends between researchers aligned. These results show that lens diameter is important when comparing the isotope values of lens laminae. The results underscore the significant impact that different individuals have on the eye lens peeling process, thereby emphasizing the need for an alternative approach to ensure quality control.

Despite the differences between researchers, the use of lamina number is still a valid method to answer exploratory questions that can lead to more in-depth research, as it can provide preliminary lifelong diet information when measuring tools are unavailable or too costly. Consistency was observed within researchers, suggesting that lamina number may be used if there is one person consistently delaminating lenses, especially when the study aims to identify shifts in one individual’s diet over time. Yet, caution should be taken when comparing results over time and between researchers. Categorizing by lamina number can also be a useful naming system for keeping individual lamina organized for sample submission, storage, and in a database. Delaminating generates many samples per individual fish; having a consistent naming system, such as lamina number is important for data organization.

Lens diameter offers a more dependable marker for growth categorization, overcoming the limitations associated with the use of lamina number. The adoption of lens diameter corrects for biases introduced by human effects that are tied to lamina number. Using lens diameter instead of lamina number as a comparison metric enhances the reliability and reproducibility of lens delamination. Additionally, it allows for the evaluation of results between different studies, offering a universal baseline for comparison.

Future works

A film of gelatinous material is observed on the surface of most lenses. Often referred to as the outer cortex, this coating is likely a partially formed lamina that has not yet compressed into the hardened crystallin structure that the rest of the lens exhibits [2,13]. Obtaining an accurate measurement of the outer cortex's diameter is challenging due to its irregular shape and uneven edges, which may result from the extraction or freezing process. For the purposes of this methodology paper, the outer cortexes were carefully removed before the lenses were measured by rolling them on clean Kimwipes. Despite the challenges, developing standardized methods to measure the outer cortex is a valuable endeavor that will aid our understanding in the life history of fish and serve as a crucial component in establishing the relationship between fork length and lens diameter.

Acknowledgment

We extend our gratitude to Paloma Herrera-Thomas for her exceptional illustration of Chinook Salmon and its lens. Additionally, we would like to thank the Stable Isotope Facility (SIF) at the University of California, Davis, for conducting the isotope analyses on all our samples.

References:

1. Young MJ, Larwood V, Clause JK, Bell-Tilcock M, Whitman G, Johnson R, et al. Eye lenses reveal ontogenetic trophic and habitat shifts in an imperiled fish, Clear Lake hitch (Lavinia exilicauda chi). Canadian Journal of Fisheries and Aquatic Sciences. 2022;79: 21–30. doi:10.1139/cjfas-2020-0318

2. Leifsdóttir RR, Campana SE. Species independence of eye lens dimensions in teleosts and elasmobranchs. PLoS One. 2023;18. doi:10.1371/journal.pone.0286388

3. Wallace AA, Hollander DJ, Peebles EB. Stable isotopes in fish eye lenses as potential recorders of trophic and geographic history. PLoS One. 2014;9. doi:10.1371/journal.pone.0108935

4. Quaeck-Davies K, Bendall VA, MacKenzie KM, Hetherington S, Newton J, Trueman CN. Teleost and elasmobranch eye lenses as a target for life-history stable isotope analyses. PeerJ. 2018;2018. doi:10.7717/peerj.4883

5. Bell-Tilcock M, Jeffres CA, Rypel AL, Sommer TR, Katz JVE, Whitman G, et al. Advancing diet reconstruction in fish eye lenses. Methods Ecol Evol. 2021;12: 449–457. doi:10.1111/2041-210X.13543

6. Tzadik OE, Curtis JS, Granneman JE, Kurth BN, Pusack TJ, Wallace AA, et al. Chemical archives in fishes beyond otoliths: A review on the use of other body parts as chronological recorders of microchemical constituents for expanding interpretations of environmental, ecological, and life-history changes. Limnol Oceanogr Methods. 2017;15: 238–263. doi:10.1002/lom3.10153

7. Trueman CN, St John Glew K. Isotopic Tracking of Marine Animal Movement. In: Hobson KA, Wassenaar LIBT-TAM with SI (Second E, editors. Tracking Animal Migration with Stable Isotopes. Academic Press; 2019. pp. 137–172. doi:10.1016/B978-0-12-814723-8.00006-4

8. Johnson RC, Garza JC, MacFarlane RB, Grimes CB, Phillis CC, Koch PL, et al. Isotopes and genes reveal freshwater origins of Chinook salmon Oncorhynchus tshawytscha aggregations in California's coastal ocean. Mar Ecol Prog Ser. 2016;548. doi:10.3354/meps11623

9. Hobson K, Wassenaar L. Tracking animal migration with stable isotopes. Terrestrial Ecology Series 2. 2008.

10. Bell-Tilcock M, Jeffres CA, Rypel AL, Willmes M, Armstrong RA, Holden P, et al. Biogeochemical processes create distinct isotopic fingerprints to track floodplain

rearing of juvenile salmon. PLoS One. 2021;16: 1–23. doi:10.1371/journal.pone.0257444

11. Bastos RF, Condini M V., Barbosa EF, Oliveira RL, Almeida LL, Garcia AM, et al. Seeing further into the early steps of the endangered atlantic goliath grouper (Epinephelus itajara): Eye lenses high resolution isotopic profiles reveal ontogenetic trophic and habitat shifts. Mar Environ Res. 2024;198: 106517. doi:10.1016/j.marenvres.2024.106517

12. Zhang J, Liu B, Hu S, Chen X, Zhang X. Ontogenetic Trophic Shifts by Ommastrephes bartramii in the North Pacific Ocean Based on Eye Lens Stable Isotopes. Fishes. 2022;7. doi:10.3390/fishes7050295

13. Fernald RD, Wright SE. Maintenance of optical quality during crystalline lens growth. Nature 1983 301:5901. 1983;301: 618–620. doi:10.1038/301618a0

14. Team RC. R: A Language and Environment for Statistical Computing (version 3.4.4). Vienna, Austria: R Foundation for Statistical Computing; 2018. Available: https://www.r-project.org/

15. Wallace AA, Ellis GS, Peebles EB. Reconstructions of individual fish trophic geographies using isotopic analysis of eyelens amino acids. PLoS One. 2023;18: 1–14. doi:10.1371/journal.pone.0282669

16. Granneman JE. Evaluation of trace-metal and isotopic records as techniques for tracking lifetime movement patterns in fishes Scholar Commons Citation. ProQuest Dissertations and Theses. University of South Florida. 2018. Available: https://scholarcommons.usf.edu/etd

17. Meath B, Peebles EB, Seibel BA, Judkins H. Stable isotopes in the eye lenses of Doryteuthis plei (Blainville 1823): Exploring natal origins and migratory patterns in the eastern Gulf of Mexico. Cont Shelf Res. 2019;174: 76–84. doi:10.1016/j.csr.2018.12.013

Supplemental Information

Figure S1: Mass (mg) of each lamina plotted against lamina number. Laminae were weighed using a microbalance. Researcher 1 (orange) peeled fewer and heavier lamina on average. Researcher 2, however, tended to peel lighter lamina, resulting in more lamina.

Table S1: Analysis of Variance (ANOVA) on Lens Diameter Variation by Lamina Number and Researcher. Summary of the ANOVA results for the linear model assessing the effects of lamina number, researcher, and their interaction on lens diameter. Significant F-values indicate strong effects for each variable and their interaction. Statistical significances are provided, with *** indicating $p < 0.001$.

Figure1

Click here to access/download;Figure;Fig1.tif

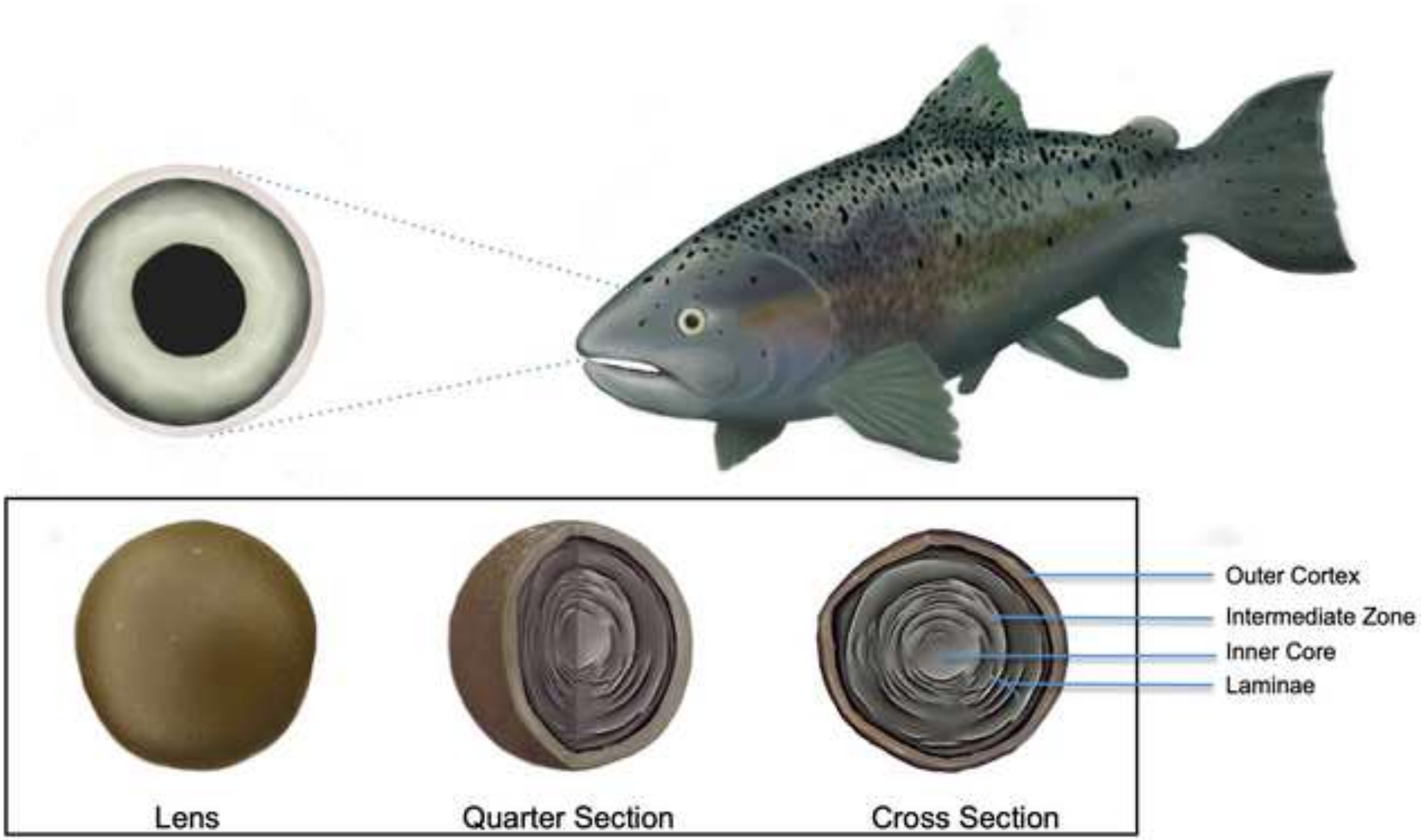

Figure2

Click here to access/download;Figure;Fig2.tiff

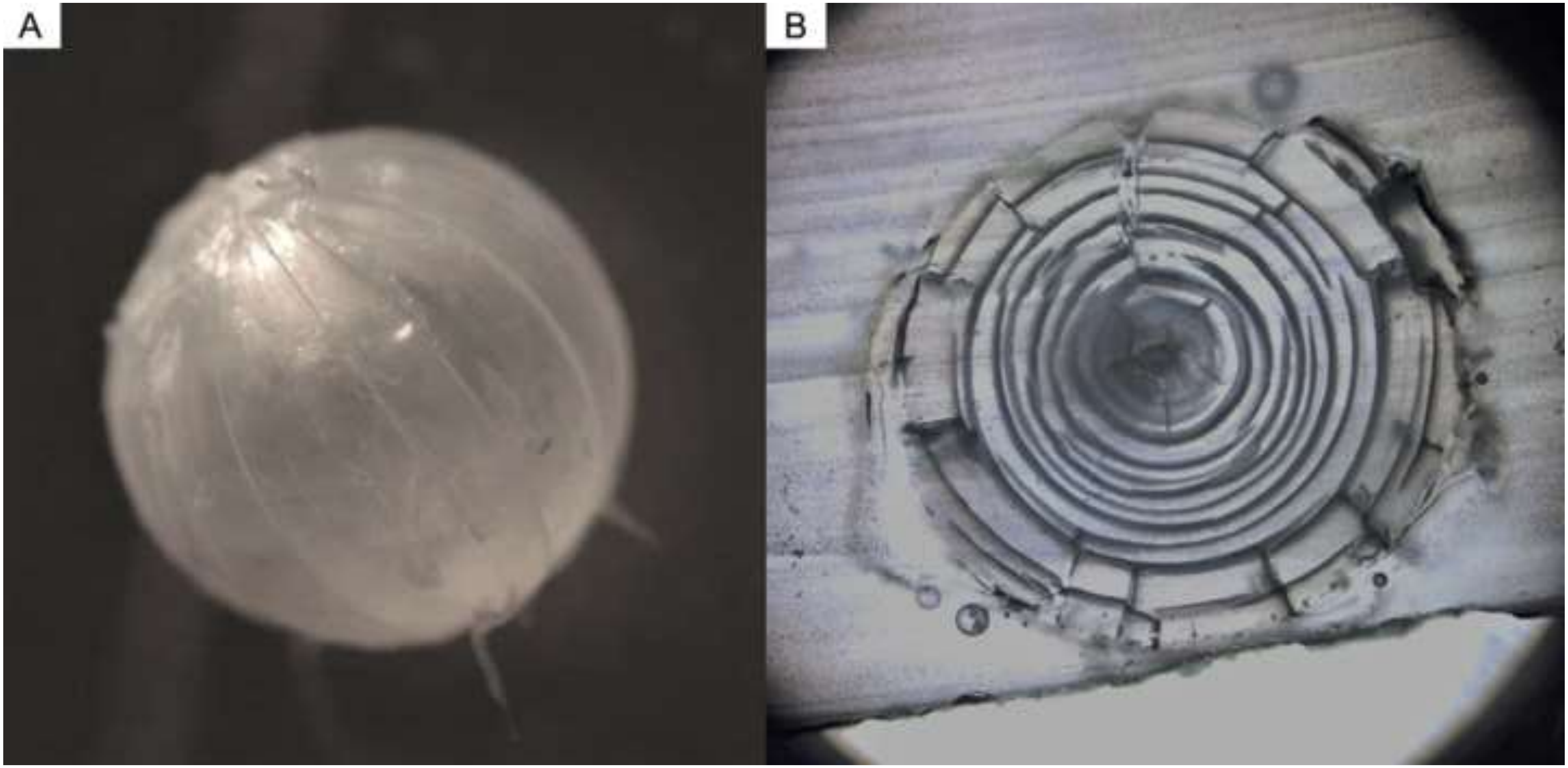

Figure3

Click here to access/download;Figure;Fig3.tif

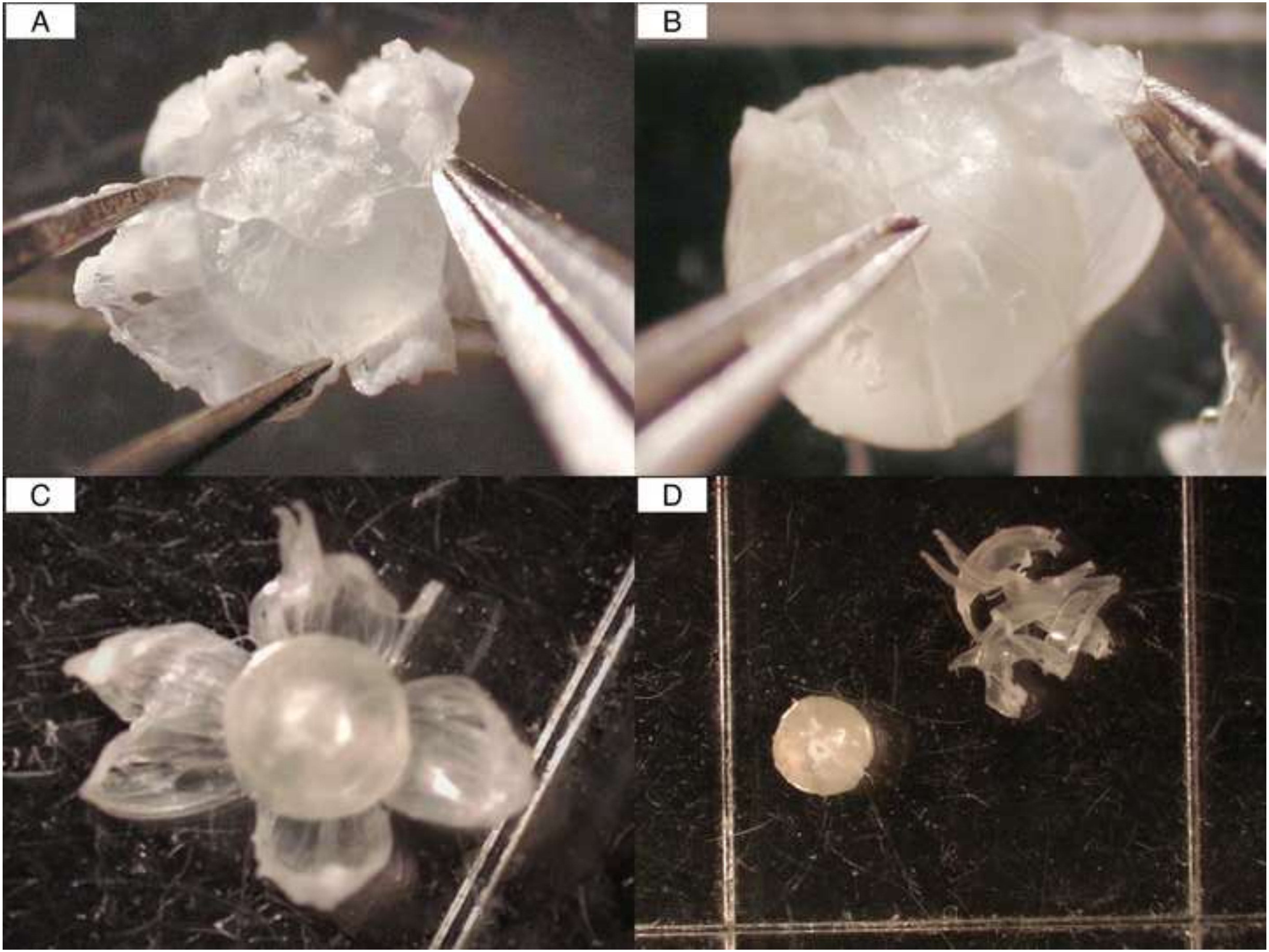

Click here to access/download;Figure;Fig4.tiff

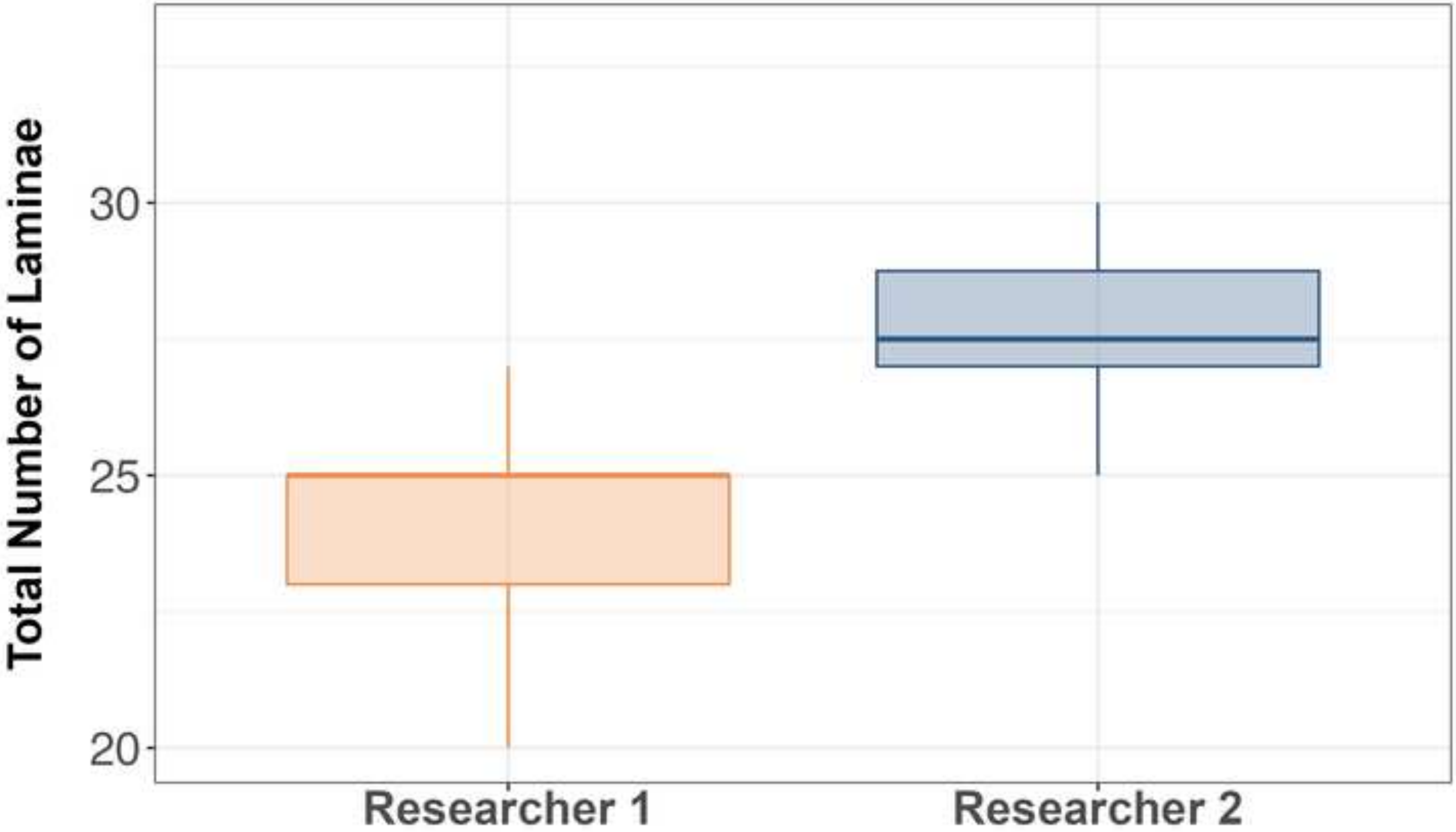

Click here to access/download;Figure;Fig5.tiff

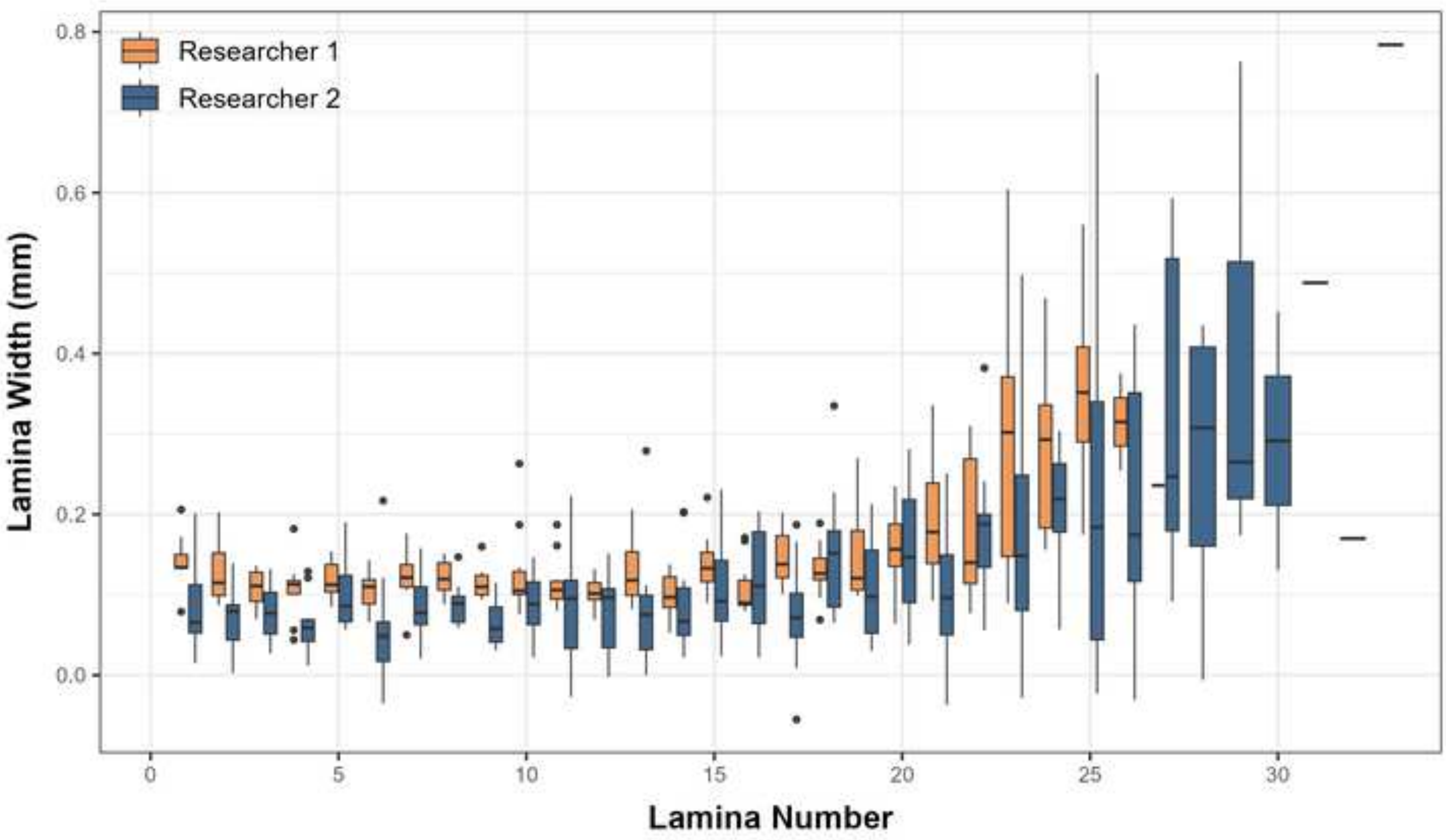

Figure6

Click here to access/download;Figure;Fig6.tiff

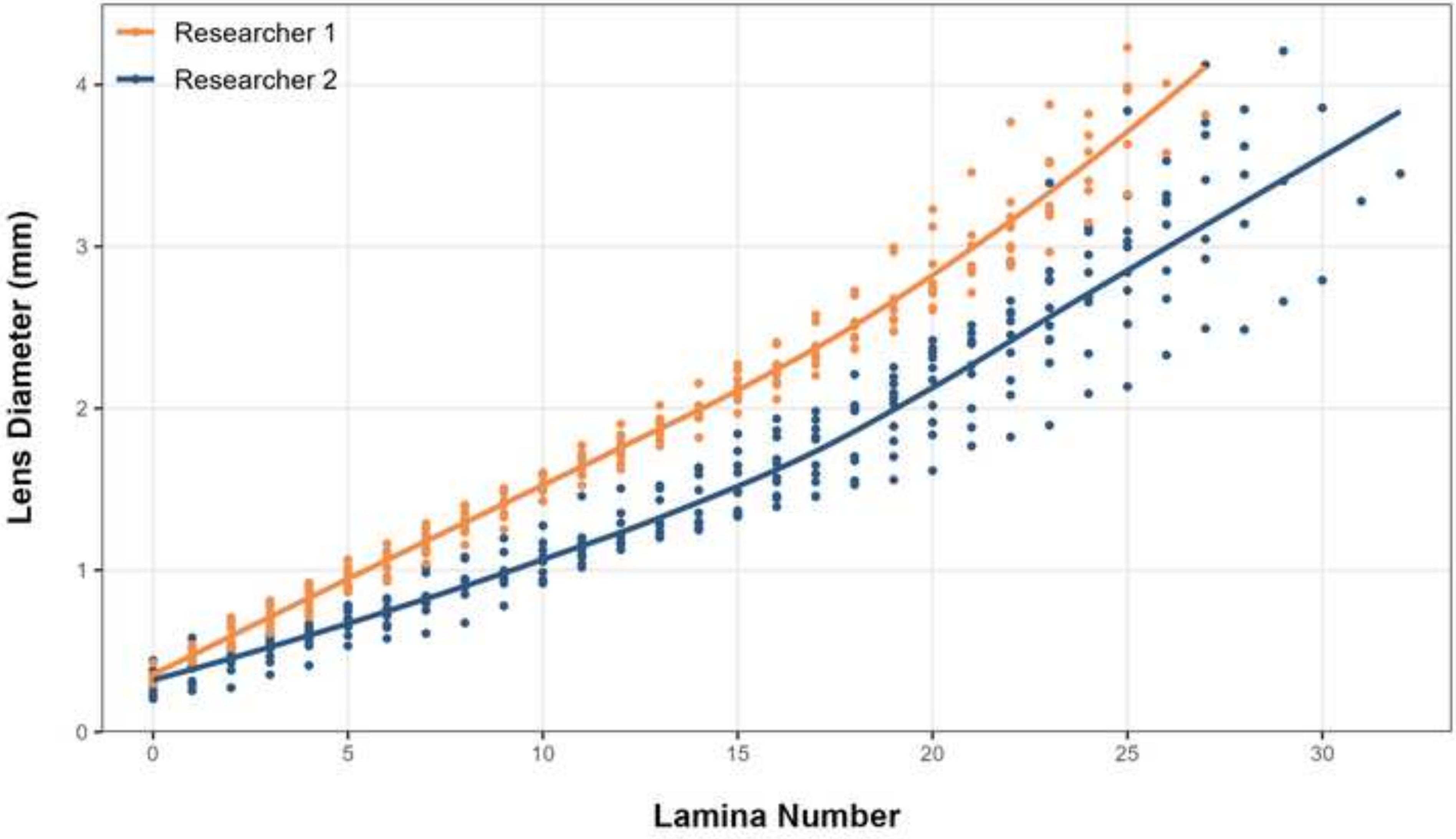

Figure7

Click here to access/download;Figure;Fig7.tiff

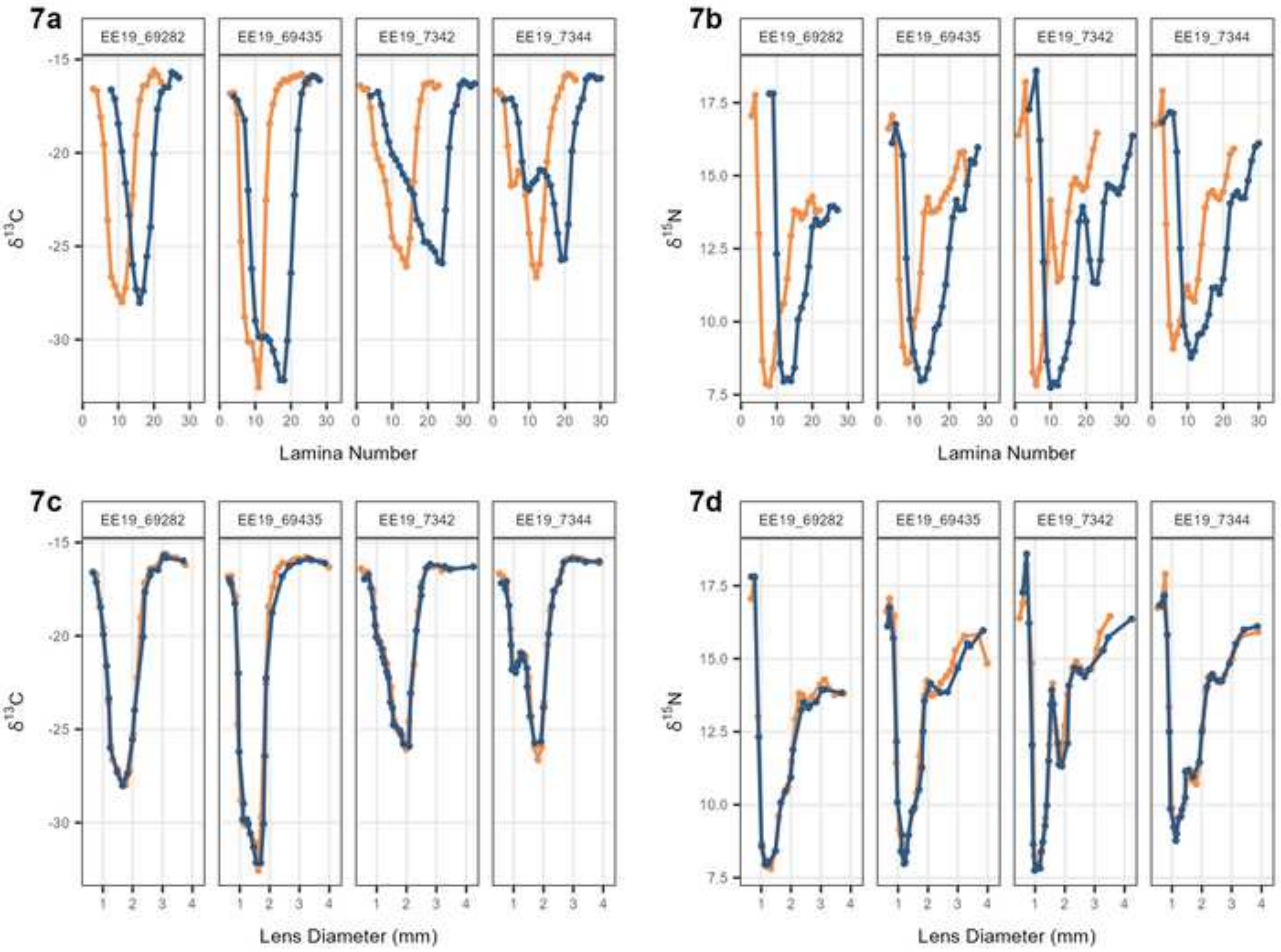

FigureS1

Click here to access/download;Figure;FigS1.tiff

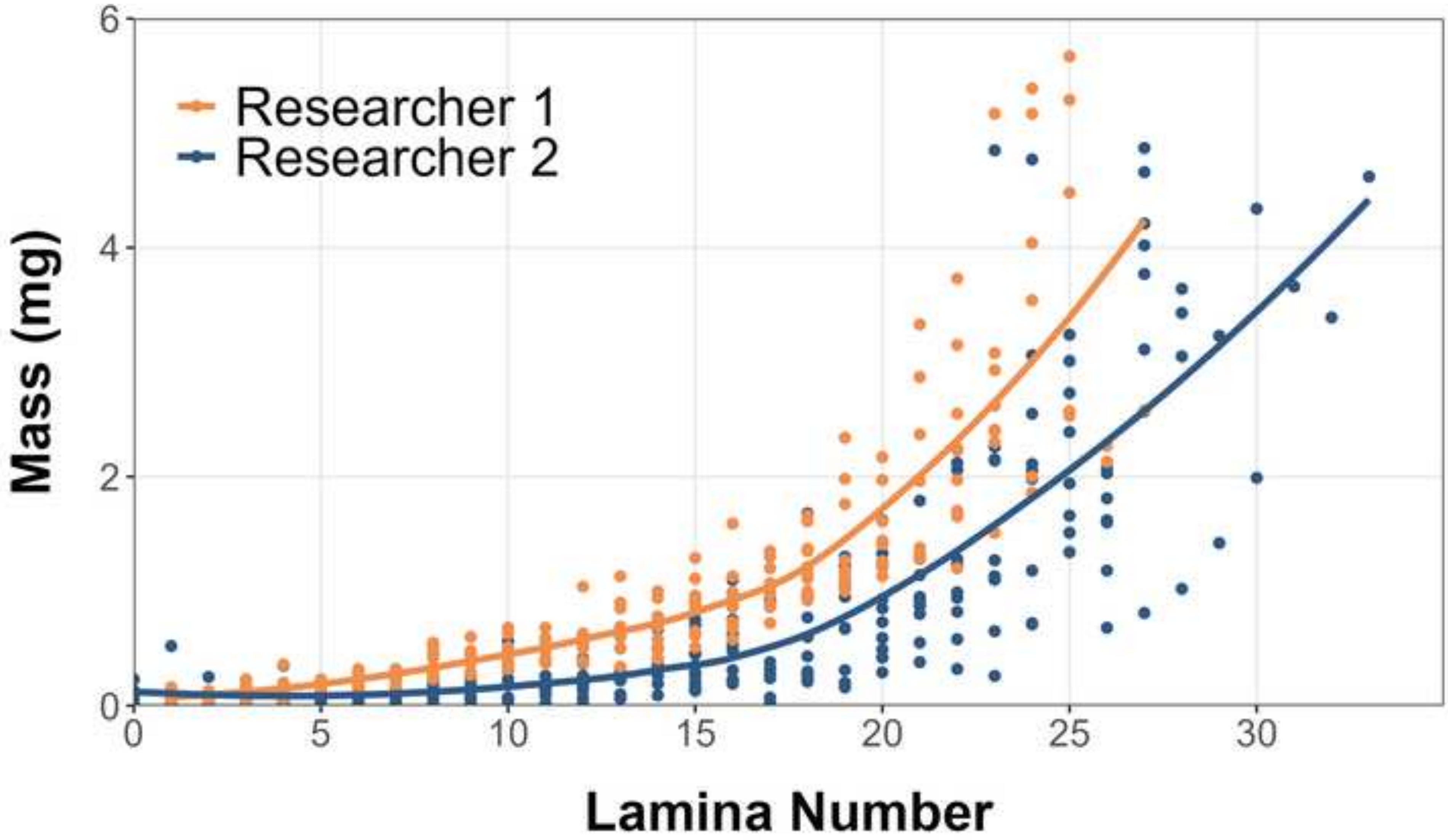

Click here to access/download
Supporting Information - Compressed/ZIP File Archive
S1_appendix.docx
